# Very Early Versus Early Percutaneous Coronary Intervention in Patients with Decreased e-GFR after Successful Fibrinolytic Therapy

**DOI:** 10.5334/gh.794

**Published:** 2020-04-16

**Authors:** Mohamed Khalfallah, Randa Abdelmageed, Amany Allaithy

**Affiliations:** 1 Department of Cardiovascular Medicine, Faculty of Medicine, Tanta University, EG

**Keywords:** fibrinolytic therapy, STEMI, pharmacoinvasive strategy, percutaneous coronary intervention, contrast-induced nephropathy

## Abstract

**Background:**

Pharmacoinvasive strategy (PIS) is the alternative approach to primary percutaneous coronary intervention (PCI) if PCI capable center isn’t available especially in the developing countries. Our objective of the current study was to investigate the incidence of contrast induced nephropathy (CIN), the occurrence of no reflow phenomenon and major adverse cardiac events (MACE) in patients with decreased estimated glomerular filtration rate (e-GFR) after successful fibrinolytic therapy in order to assess the benefit from very early PCI strategy (within 3–12 hours) or early PCI strategy (within 12–24 hours).

**Methods:**

This randomized clinical trial included 420 patients with STEMI. All participants were classified randomly into two groups according to the time of intervention; Group I patients were subjected to very early PCI (within 3–12 hours) and Group II patients were subjected to early PCI (within 12–24 hours) after receiving successful fibrinolytic therapy.

**Results:**

The incidence of CIN in Group I was slightly higher than Group II (23 patients [10.7%] versus 19 patients [9.3%]) respectively, with no statistically significant difference between the two groups (P value = 0.625). The incidence of no-reflow phenomenon (TIMI 0–2 flow) after the procedure was higher in Group II, while TIMI 3 flow (normal flow) was significantly higher in Group I than Group II (184 [85.6%] vs. 153 [74.6%], respectively) with P value = 0.044. There was no statistically significant difference between the two groups regarding mortality and MACE.

**Conclusion:**

The incidence of CIN was nearly equal in very early PCI (within 3–12 hours) versus early PCI (within 12–24 hours); however, the incidence of no-reflow phenomenon was significantly higher in patients subjected to early PCI (within 12–24 hours).

## Introduction

The ideal method of reperfusion in patients with acute ST elevation myocardial infarction (STEMI) according to the recent guidelines is primary percutaneous coronary intervention (PPCI) [1,2]. In real practice, especially in developing countries, PPCI is not always applicable due to unavailability of percutaneous coronary intervention (PCI) capable centers, so the alternative approach is to give fibrinolytic therapy followed by either rescue PCI for patients with failed fibrinolysis or non-immediate PCI after successful fibrinolysis within 3 to 24 hours, and this is known as pharmacoinvasive strategy (PIS) [3,4].

Acute kidney injury (AKI) is a potentially reversible condition that occurs following the exposure to iodinated contrast media during PCI, and this is defined as contrast-induced nephropathy (CIN) [5]. CIN is a serious complication of coronary intervention, CIN is incriminating in increasing risk of hemodialysis and mortality [6,7,8,9]. Minor elevation of serum creatinine level leads to increased length of hospital stay and increased financial costs, so detection and prevention of CIN is considered a major health concern [10,11]. CIN is defined as increase in serum creatinine level by ≥0.5 mg/dl above the admission value or a >25% relative rise during the first 72 hours after the procedure [12]. The incidence of CIN after PPCI is increasing due to the emergency condition, as there is no time for implementation of prophylactic measures against CIN in high-risk patients and dealing with hemodynamic compromise [13].

The objective of the present study was to investigate the incidence of CIN, the occurrence of no-reflow phenomenon and major adverse cardiac events (MACE) in patients with decreased estimated glomerular filtration rate (e-GFR) who were subjected to PIS in order to assess the benefit from very early PCI strategy (within 3–12 hours) after successful fibrinolytic therapy followed by intravenous hydration by normal saline for 24 hours. The study questioned whether it is better to hydrate the patients first for 12 hours before PCI to decrease the potential harmful effects of contrast media and to control the hemodynamics of the patients. The patients were subjected to early PCI strategy (within 12–24 hours) to be followed by another 12 hours of intravenous hydration after PCI.

## Patients and Methods

This is a randomized clinical trial included patients with STEMI with decreased e-GFR, who were managed by PIS. The patients were transferred to our cardiovascular department from other surrounding hospitals after receiving successful fibrinolytic therapy and referred to our hospital for PCI as a part of PIS during the period from January 2017 to January 2019.

### Study protocol

The study was conducted on 420 patients with STEMI with e-GFR >90 mL/min/1.73 m^2^ who were classified randomly into two groups according to the time of intervention; Group I patients were subjected to very early PCI (within 3–12 hours) after successful fibrinolytic therapy, and Group II patients were subjected to early PCI (within 12–24 hours) after successful fibrinolytic therapy.

The included patients were randomized using a computerized random number generator to select randomly permuted blocks with a block size of five and an equal allocation ratio. Allocation concealment was performed using sequentially numbered opaque sealed envelopes, which were opened after the patient signed the written informed consent and then enrolled into the respective group. All patients gave a written informed consent, and the study was registered and approved by the Local Research Ethics Committee of the Faculty of Medicine, Tanta University, and was in accordance with the principles of the Declaration of Helsinki II.

### Exclusion criteria

Patients were excluded if they were on hemodialysis or received contrast media during the week preceding the procedure.

### Demographic, clinical and laboratory data

All patients were subjected to full history taking and clinical examination. Electrocardiogram (ECG) was done and compared to the initial ECG for signs of success of fibrinolytic therapy. Blood samples were collected for routine laboratory investigations. Patients were considered at high risk for CIN if they had chronic kidney disease (CKD) with high baseline serum creatinine, and if they were diabetic, hypertensive, anemic or elderly [14]. Serum creatinine was measured at the time of admission and repeated after 24, 48 and 72 hours. Patients with CIN were followed up for 10 days after the procedure, and serum creatinine was measured at that time. Creatinine clearance was calculated using the modification of diet in renal disease (MDRD) equation. Non-ionic iso-osmolar contrast media was used for all patients during PCI.

Patients in Group I very early PCI (within 3–12 hours) were hydrated with intravenous 0.9% saline during PCI at a rate of 1 ml/kg/h or 0.5 ml/kg/h in patients with cardiogenic shock, heart failure or left ventricular ejection fraction <40% and continued for 24 hours after the procedure. While, patients in Group II early PCI (within 12–24 hours) were hydrated by the same regimen 12 hours before the procedure and 12 hours after the procedure.

### Percutaneous coronary intervention

Patients pre-medicated according to the guidelines with the standard medications including clopidogrel 600 mg, heparin and acetyl salicylic acid 300 mg. Patients then transferred to the catheterization laboratory. Diagnostic coronary angiography was performed using radial or femoral approach for all patients. Then, the culprit lesion was determined; its location, TIMI flow, thrombus burden. Pre-dilatation was done with a balloon then a stent or stents were deployed as determined by the operators. The volume of contrast agent was adjusted according to the guidelines. Patients received medications after completing the procedure according to the latest guidelines [15]. The patients in both groups were followed up for one month for the occurrence of CIN and other MACE.

### Endpoints

The primary endpoint of the study was the occurrence of CIN, which was defined as reversible acute renal failure after exposure to iodinated contrast agent during angiography with a relative (≥25%) or absolute (≥0.5 mg/dl) increase in serum creatinine from baseline within three days after contrast media exposure that usually recovers within 7–10 days with most of the patients return to their baseline values [12]. The secondary endpoints were the occurrence of no reflow phenomenon and other MACE including cardiogenic shock, heart failure, major bleeding and mortality. No reflow phenomenon was defined as incomplete restoration of myocardial reperfusion in epicardial coronary artery after adequate reopening of the infarct related artery with TIMI flow ≤2. TIMI flow score was defined by the degree of flow into the epicardial coronary artery with the following grades: Grade 0 = complete absence of flow beyond the point of obstruction; Grade 1 = some contrast material flows distal to the obstruction, but complete arterial visualization is not achieved; Grade 2 = delayed opacification of the entire artery; and Grade 3 = full prompt visualization of the entire artery [16,17].

### Statistical analysis

Statistical analysis was done using SPSS 23, IBM, Armonk, NY, United States of America. Quantitative data were expressed as mean ± standard deviation. Qualitative data were expressed as frequency and percentage. Student’s t test of significance was used when comparing between two means. Chi-square test of significance was used to compare between two qualitative parameters. P value < 0.05 was considered statistically significant.

## Results

**Figure 1:**
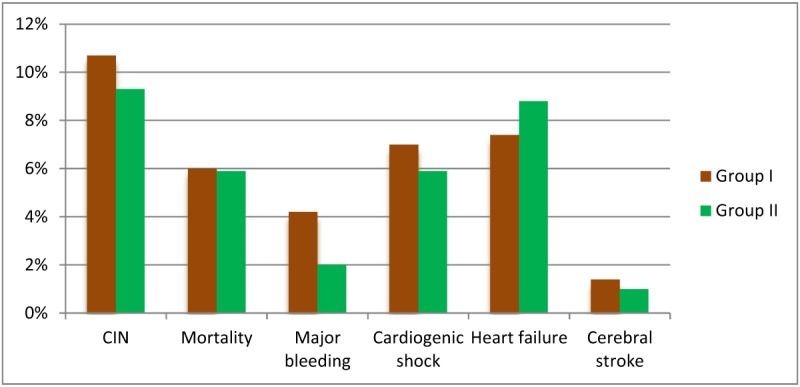
Comparison of the outcome after percutaneous coronary intervention between group I very early PCI (within 3–12 h) and group II early PCI (within 12–24 h).

The study was conducted on 420 patients with STEMI presented with e-GFR >90 mL/min/1.73 m^2^ at the time of admission. Patients were divided into two groups according to the time of intervention; Group I included 215 patients who were subjected to very early PCI (within 3–12 hours), and Group II included 205 patients who were subjected to early PCI (within 12–24 hours) after successful fibrinolytic therapy. The main finding of the present study was that the incidence of CIN in Group I was slightly higher than Group II, as there were 23 patients (10.7%) with CIN in group I versus 19 patients (9.3%) in Group II, but there was no statistically significant difference between the two groups (P value = 0.625). Regarding the demographic data, clinical characteristics and outcome, there was no statistically significant difference between both groups except diastolic blood pressure was significantly higher in patients in Group I (P value = 0.046) as shown in Table 1 and Figure 1.

**Table 1:** Demographic data, clinical characteristics, and outcome of all patients in the two groups.

	Group I (N = 215) (very early PCI)	Group II (N = 205) (early PCI)	P value

Age, years	60.01 ± 8.74	59.81 ± 8.51	0.813
Male gender, n (%)	123 (57.2%)	110(53.7%)	0.464
Hypertension, n (%)	79(36.7%)	86(42.0%)	0.275
Diabetes mellitus, n (%)	122(56.7%)	111(54.1%)	0.592
Smoking, n (%)	116(54.0%)	99(48.3%)	0.246
Dyslipidemia, n (%)	76(35.3%)	77(37.6%)	0.638
Prior myocardial infarction, n (%)	16 (7.4%)	17 (8.3%)	0.746
CKD, n (%)	27(12.6%)	26(12.7%)	0.969
BMI, (kg/m^2^)	25.1 ± 3.21	24.6 ± 3.01	0.151
Systolic BP, mmHg	117.3 ± 20.6	120.1 ± 17.9	0.143
Diastolic BP, mmHg	77.6 ± 12.3	79.9 ± 11.1	0.046*
LVEF, (%)	45.88 ± 4.78	46.55 ± 4.77	0.151
CIN, n (%)	23(10.7%)	19(9.3%)	0.625
Mortality, n (%)	13 (6.0%)	12 (5.9%)	0.933
Major bleeding, n (%)	9 (4.2%)	4 (2.0%)	0.186
Cardiogenic shock, n (%)	15(7.0%)	12(5.9%)	0.639
Heart failure, n (%)	16(7.4%)	18(8.8%)	0.615
Cerebral stroke, n (%)	3 (1.4%)	2(1.0%)	0.692

BMI: body mass index; BP: blood pressure; LVEF: left ventricular ejection fraction; CIN: contrast induced nephropathy.

**Figure 2:**
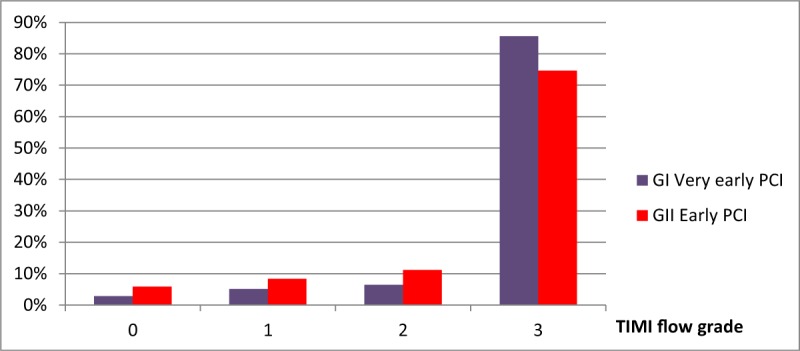
Comparison of TIMI flow after percutaneous coronary intervention between group I very early PCI (within 3–12 h) and group II early PCI (within 12–24 h).

With respect to laboratory results, there was no statistically significant difference between both groups regarding serum hemoglobin, CK MB, random blood sugar, serum creatinine and e-GFR pre and post-procedure, as shown in Table 2. Regarding the angiographic results, the study showed no statistically significant difference between both groups regarding the culprit vessel, thrombus burden or volume of contrast agent. However, the incidence of no reflow after the procedure (TIMI 0–2 flow) was higher in Group II, while the normal flow (TIMI 3 flow) was achieved in Group I better than group II (184 [85.6%] vs. 153 [74.6%] respectively) with P value = 0.044. Also the need for aspiration catheters was higher in Group II than Group I (34 [16.6%] vs. 21 [9.8%] respectively) with P value = 0.038 as shown in Table 3 and Figure 2.

**Table 2:** Laboratory results of all patients in the two groups.

	Group I (N = 215) (very early PCI)	Group II (N = 205) (early PCI)	P value

Random blood sugar, mg/dl	142.1 ± 33.1	139.6 ± 25.4	0.379
CK-MB, U/L	73.2 ± 33.1	75.8 ± 32.8	0.421
Hemoglobin level, g/dl	11.19 ± 1.68	11.39 ± 1.61	0.216
Creatinine pre-procedure, mg/dl	1.47 ± 0.36	1.45 ± 0.34	0.596
Creatinine post-procedure, mg/dl	1.54 ± 0.49	1.56 ± 0.46	0.633
E-GFR pre-procedure, (N& %)			
60–89 (mL/min/1.73 m^2^)	109 (50.7%)	110(53.7%)	0.718
30–59 (mL/min/1.73 m^2^)	89 (41.4%)	77(37.6%)	
<30 (mL/min/1.73 m^2^)	17 (7.9%)	18 (8.8%)	
E-GFR post-procedure, (N& %)			
60–89 (mL/min/1.73 m^2^)	86 (40.0%)	99 (48.3%)	0.152
30–59 (mL/min/1.73 m^2^)	106 (49.3%)	82 (40.0%)	
<30 (mL/min/1.73 m^2^)	23 (10.7%)	24 (11.7%)	
E-GFR pre-procedure,(M ± SD)			
60–89 (mL/min/1.73 m^2^)	77.50 ± 7.96	78.32 ± 7.13	0.418
30–59 (mL/min/1.73 m^2^)	46.27 ± 9.19	45.59 ± 9.04	0.634
<30 (mL/min/1.73 m^2^)	28.18 ± 0.78	27.59 ± 1.22	0.103
E-GFR post-procedure,(M ± SD)			
60–89 (mL/min/1.73 m^2^)	74.24 ± 8.62	76.05 ± 8.08	0.144
30–59 (mL/min/1.73 m^2^)	42.86 ± 8.15	43.52 ± 8.15	0.585
<30 (mL/min/1.73 m^2^)	27.61 ± 1.99	27.10 ± 1.51	0.332

CK-MB: Creatine kinase myocardial band; E-GFR: estimated glomerular filtration rate.

**Table 3:** Angiographic results of all patients in the two groups.

	Group I (N = 215) (very early PCI)	Group II (N = 205) (early PCI)	P value

Culprit vessel			
LM coronary artery, n (%)	3(1.4%)	2(1.0%)	0.692
LAD coronary artery, n (%)	88(40.9%)	79(38.5%)	0.616
CX coronary artery, n (%)	62(28.8%)	69(33.7%)	0.286
Right coronary artery, n (%)	61(28.4%)	56(27.3%)	0.809
Thrombus burden			
Low	71 (33.0%)	74 (36.1%)	0.620
Moderate	74 (34.4%)	73 (35.6%)	
High	70 (32.6%)	58 (28.3%)	
Aspiration catheter	21 (9.8%)	34 (16.6%)	0.038*
Reperfusion type			
Balloon angioplasty	16(7.4%)	7 (3.4%)	0.193
Direct stenting	62(28.8%)	61 (29.8%)	
Stenting after pre-dilatation	137 (63.7%)	137(66.8%)	
Post-procedural TIMI flow			
0	6 (2.8%)	12 (5.9%)	0.044*
1	11 (5.1%)	17 (8.3%)	
2	14 (6.5%)	23 (11.2%)	
3	184 (85.6%)	153(74.6%)	
Volume of contrast agent,(ml)	131.4 ± 45.68	125.4 ± 35.62	0.134

LM: left main; LAD: left anterior descending; CX: circumflex; TIMI: thrombolysis in myocardial infarction.

## Discussion

Great attention was given to CIN in recent years as being a dreadful complication of coronary intervention. CIN is a potentially avoidable condition, but when develops, it leads to increased morbidity and mortality [5,6,7,8,9]. In the current study we hypothesized that CIN will be less prevalent in patients who were subjected to early PCI strategy (within 12–24 hours) due to adequate intravenous hydration given to the patients 12 hours before the procedure and better hemodynamics control. We found that the incidence of CIN in Group II (early PCI within 12–24 hours) was slightly lower than Group I (very early PCI within 3–12 hours) (19 [9.3%] vs. 23 [10.7], respectively). However, there was no statistically significant difference between both groups (P value = 0.625). The incidence of CIN varies greatly after coronary intervention; it may be as low as 6% in patients undergoing elective catheterization and it reaches 25% in urgent catheterization [18,19]. In agreement with our results, Ivanes et al. [5], who studied the predictive factors of CIN in patients undergoing primary coronary angioplasty found that the incidence of CIN was 9.1% in such patients.

Contrast-induced nephropathy implies impairment of renal function occurring within three to five days after contrast media administration in the absence of an alternative etiology [20]. However, contrast media isn’t the sole factor responsible for renal impairment in patients with STEMI undergoing PCI. Hypotension, hemodynamic instability and acute heart failure are additional risk factors that may lead directly to AKI or potentiate the side effects of contrast media [19]. Consequently, acute renal impairment in the setting of STEMI likely has alternative and multiple etiologies. Prevention of contrast media–associated nephropathy was investigated previously in many trials; Mueller et al. [21], who studied two hydration regimens in 1620 patients undergoing coronary angioplasty in randomized manner, reported that CIN was significantly reduced with isotonic (0.7%, 95% CI, 0.1%–1.4%) versus half-isotonic (2.0%, 95% CI, 1.0%–3.1%) hydration (P = 0.04) and the incidence of cardiac complications was similar between the two hydration groups. In the current study we also noticed the overall mortality and MACE was similar in both groups except the incidence of no-reflow was higher in Group II (early PCI within 12–24 hours).

No-reflow phenomenon is defined as incomplete restoration of myocardial reperfusion in epicardial coronary artery after adequate reopening of the infarct related artery [16]. No-reflow phenomenon can occur in up to one third of patients after PCI according to the recent studies [16,22]. The pathophysiology of no-reflow is complex and multifactorial, several mechanisms have been recognized: (1) presence of microvascular dysfunction, (2) ischemic injury, (3) reperfusion injury, (4) distal micro-thrombo-embolization and (5) individual susceptibility [23,24,25]. Myocardial necrosis occurs within six hours of acute coronary occlusion, so prolonged ischaemia leads to distal capillary bed edema and myocardial bed swelling. Furthermore, obliteration of coronary lumen by neutrophil-platelet plugging that leads to coronary microvascular dysfunction and obstruction. Moreover, production of large amounts of vasoconstrictors and inflammatory mediators, endothelial cells release adhesion molecules, e.g. intercellular adhesion molecule-1 with oxygen free radicals release and endothelin, which contribute to the pathogenesis of no-reflow phenomenon [23,24,25].

In the early stages of acute coronary occlusion, thrombus is friable and rich in thrombocytes and it is easier to be treated with adjunctive pharmacrotherapy. However, with delayed reperfusion, the thrombus becomes more rigid, and after its fragmentation may lead to distal micro-thrombo-embolization. Delayed reperfusion results in a well-organized thrombus which reduces the likelihood of achievement of TIMI 3 flow [26,27].

In the current study, although the fibrinolytic therapy was successful in all patients, we found that Group I (very early PCI within 3–12 hours) achieved TIMI 3 flow higher than Group II (early PCI within 12–24 hours). Although fibrinolytic therapy achieved flow firstly and opened the occluded artery, the residual thrombi in the vessel will be more organized with delayed intervention, with loss of its friability, and this can lead to the no-reflow phenomenon after coronary intervention. Also, re-occlusion of the infarct related artery after fibrinolytic therapy can occur more frequently with delayed intervention. Consequently, more myocardial damage occurs and the incidence of no-reflow phenomenon will be more frequent.

## Conclusion

The incidence of CIN wasn’t significantly different in both regimens of treatment. Patients with STEMI with decreased e-GFR who received successful fibrinolytic therapy should be subjected to very early PCI (within 3–12 hours) followed by intravenous hydration with 0.9% saline whatever the baseline serum creatinine for better achievement of TIMI 3 flow. Mortality and MACE were equal between the two regimens of treatment; however, no-reflow phenomenon was significantly higher in patients subjected to early PCI (within 12–24 hours).

## Study limitations

1. The patients were enrolled at a single center, which does not reflect the whole population.
2. The number of the patients in this study was relatively small, so additional multicenter studies with larger population numbers will validate the results.
3. The assessment of no-reflow phenomenon was angiographically defined in this study. Pre- and post-procedural assessment of the culprit lesion by intravascular ultrasound (IVUS) to assess the cause and mechanisms of no-reflow phenomenon including multiple ruptured plaques, plaques with high thrombus burden and plaque prolapse after stenting wasn’t performed due to financial costs.
4. There are variable definitions of CIN have been used in studies evaluating the effects of different contrast media and strategies to prevent CIN, so the incidence of CIN accordingly is variable. Moreover, the development of acute kidney injury may occur in patients with STEMI undergoing PCI due to other causes rather than the use of contrast media including acute heart failure and hemodynamic instability.
5. Long-term mortality and MACE were not detected, as we followed the patients only for one month after PCI.

## Future Directions

In spite of these limitations, the study opened the door for further multicenter randomized controlled trials to validate the results with a large proportion of population. Pharmacoinvasive strategy is considered the cornerstone in management of STEMI especially in developing countries with few PCI-capable centers.
